# SOX21-AS1 activated by STAT6 promotes pancreatic cancer progression via up-regulation of SOX21

**DOI:** 10.1186/s12967-022-03521-5

**Published:** 2022-11-05

**Authors:** Dandan Yu, Zhigang Zhao, Li Wang, Shishi Qiao, Zhen Yang, Qiang Wen, Guanghui Zhu

**Affiliations:** 1 Medical Oncology Department, Zhengzhou Yihe Hospital, Henan University, Shanghai, China; 2 Zhengzhou Yihe Hospital Affiliated to Henan University, Shanghai, China; 3 Hepatobiliary and Pancreatic Surgery, The First Affiliated Hospital of Zhengzhou University, Shanghai, China; 4 Gastrointestinal Surgery, The First Affiliated Hospital of Zhengzhou University, Shanghai, China; 5 Institute of Clinical Pharmacology, Zhengzhou University, Shanghai, China; 6 Department of Gastrointestinal Surgery, Shanghai Municipal Hospital of Traditional Chinese Medicine, Shanghai University of Traditional Chinese Medicine, No.274, Zhijiang Middle Road, Jing’an District, Shanghai, China

**Keywords:** Pancreatic cancer, SOX21-AS1, SOX21, STAT6

## Abstract

**Background:**

Pancreatic cancer (PC) is a highly malignant tumor which threatens human’s health. Long non-coding RNAs (lncRNAs) are implicated in many cancers, including PC, but their mechanisms in PC have not yet been entirely clarified. We focused on revealing the potential function of lncRNA SOX21-AS1 in PC.

**Methods:**

Functional assays assessed SOX21-AS1 function on PC progression. Bioinformatics analysis, along with mechanism assays were taken to unmask the regulatory mechanism SOX21-AS1 may exert in PC cells.

**Results:**

SOX21-AS1 possessed a high expression level in PC cells. SOX21-AS1 absence suppressed PC cell proliferation, migration, stemness and epithelial-mesenchymal transition (EMT) while elevated cell apoptosis. SOX21-AS1 positively regulated its nearby gene SRY-box transcription factor 21 (SOX21) at post-transcriptional level. Through mechanism assays, we uncovered that SOX21-AS1 sponged SOX21-AS1 to elevate SOX21 mRNA and recruited ubiquitin-specific peptidase 10 (USP10) to deubiquitinate and stabilize SOX21 protein. Moreover, signal transducer and activator of transcription 6 (STAT6) could transcriptionally activate SOX21-AS1 and SOX21 expression.

**Conclusions:**

SOX21-AS1 aggravated the malignant development of PC, which might provide the utility value for PC treatment.

**Supplementary Information:**

The online version contains supplementary material available at 10.1186/s12967-022-03521-5.

## Background

Pancreatic cancer (PC) is an aggressive malignant tumor with high lethality [1]. It is estimated that the mortality of patients with PC is about 40,000 cases [2]. The prognosis and clinical outcomes of patients with PC are poor. Up to now, the major treatment methods for PC are surgical operation for early detection and chemotherapy [3]. In spite of various genetic and epigenetic changes recognized in PC, the accurate pathogenesis of PC remains indistinct [4]. Hence, understanding the occurrence and development of PC may be beneficial for us to identify novel and effective diagnostic and therapeutic targets for PC.

As far as we know, at least 90% of the mammalian genome is transcribed as non-coding RNAs (ncRNAs). Accumulating studies have demonstrated that these ncRNAs are not transcriptional noise due to their important functions [5]. Long non-coding RNAs (lncRNAs), as a class of ncRNA, possess the length of over 200 nucleotides [6]. Recent evidences have proved that lncRNA modulates gene expression via various mechanisms [7]. The majority of well-studied lncRNAs are found to be important modulators in affecting cellular processes including cell cycle, growth, and apoptosis which make sure homeostasis [8]. As reported previously, lncRNAs can act as oncogenes or tumor repressors to regulate the development of PC [9]. LncRNA-H19 facilitates PC cell proliferation via modulating miR-194 and PFTAIRE protein kinase 1 (PFTK1) [10]. LncRNA PXN antisense RNA 1, namely PXN‐AS1 expresses at a low level in PC and inhibits PC progression [11]. Additionally, lncRNAs regulate genes activities via multiple mechanisms [12]. In parallel, lncRNAs also can participate in cancer regulation by acting as competing endogenous RNAs (ceRNAs) to competitively bind to microRNAs (miRNAs), thereby modulating the expression of miRNAs targets at post-transcriptional levels [13]. Up to now, there are still some unknown lncRNAs in PC to be further investigated.

LncRNA SRY-box transcription factor 21 antisense divergent transcript 1 (SOX21-AS1) has been registered to exert regulatory functions in many types of cancer. SOX21-AS1 promotes breast cancer progression through the PI3K/AKT signaling pathway [14]. SOX21-AS1 aggravates glioma cell proliferation as well as cell invasion through elevating p21-activated kinase (PAK7) expression [15]. SOX21-AS1 accelerates the tumorigenesis of colorectal cancer by affecting myosin VI (MYO6) expression [16]. Moreover, SOX21-AS1 targets miR-24-3p and PIM2 to modulate lung cancer [17]. SOX21-AS1 serving as a diagnostic biomarker in cancer development has been demonstrated by many documents [18, 19]. However, how it may exert certain impact on PC is unclear.

Through our investigation, SOX21-AS1 with high expression was firstly verified in PC cells. Therefore, we were intended to verify the detailed biological function as well as the potential mechanism SOX21-AS1 may have in PC.

## Methods

### Cell culture

ATCC (Manassas, VA, USA) supplied PC cells including CFPAC-1, Capan-1, BxPc3, PANC-1 and SW1990. Human normal pancreatic duct epithelial cells HPDE6-C7 were purchased from Shanghai Huzhen Biotechnology Co., LTD (Shanghai, China). CFPAC-1 and Capan-1 cells were grown in Iscove’s Modified Dulbecco's Medium (Gibco, USA). BxPc3 cells were grown in RPMI-1640 Medium (Gibco). PANC-1 and HPDE6-C7 cells were grown in Dulbecco's Modified Eagle's Medium (Gibco). SW1990 cells were grown in Leibovitz's L-15 Medium (Gibco). All the cells were supplemented with 10% fetal bovine serum (FBS), and the culture condition was set as: 37 °C, 5% CO_2_.

### Cell transfection

Two specific shRNAs were transfected into cells to stably silence SOX21-AS1 expression, and negative control (shRNA), pcDNA, and pcDNA-SOX21 overexpression vector, miR-576-5p mimics and NC mimics, miR-576-5p inhibitor and NC inhibitor, sh-STAT6#1/2 and shRNA were synthesized by Genepharma (Shanghai, China). The plasmids transfections were used Lipofectamine 3000 (Invitrogen, USA) for 48 h.

### Quantitative reverse transcription real-time polymerase chain reaction (RT-qPCR)

Total RNA was extracted from cells and tissues using Trizol reagent (Invitrogen, USA). Then RNA reverse transcription was applied via a PrimeScript RT master mix (Takara, Japan), followed by qPCR using SYBR Premix Ex TaqTM II (Takara, Japan). GAPDH and U6 were internal references. The gene expression was calculated using the 2^−ΔΔCt^ method. The experiment was subject to three independent repeats.

### Colony formation

600 cells were grown in plates for 14 days. Then cells were washed, fixed and subsequently stained. The colony numbers more than 50 cells were counted. The experiment was in triplicate.

### 5-Ethynyl-2’-deoxyuridine (EdU)

The EdU (Ribobio, Guangzhou China) proliferation assay was performed conforming to the guidance. Cells were seeded into plates, and 100 μl medium containing 50 μM EdU was added. Then cells were fixed and counterstained by DAPI. Images were subject to the observation through a fluorescence microscopy (Nikon, Japan). The experiment was in triplicate.

### Terminal-deoxynucleoitidyl Transferase Mediated Nick End labeling (TUNEL)

Cells on slides were fixed, washed and permeabilized, followed by adding TUNEL reagent (12,156,792,910, Roche, Basel, Switzerland). DAPI was used to counterstain the cells and images were observed under a fluorescence microscope. The experiment was in triplicate.

### Flow cytometry

Transfected PC cells were plated into 6-well plates for flow cytometry with Annexin V-FITC/PI double staining kit (Invitrogen) based on the guidance of suppler. Cells were double-stained in the darkroom for 15 min, and then subject to flow cytometer for cell apoptosis analysis.

### Transwell

8 μm transwell inserts were utilized in this assay. Cells (1 × 10^5^) were cultured in a 200 μL serum-free medium and placed into the upper chamber. 600 μL of 10% FBS medium was added to the bottom chamber. After 24 h incubation, the non-migrated cells in the upper chamber were wiped and the migrated cells in the lower chamber were fixed and stained. The number of migrated cells was counted in five random views. The experiment was in triplicate.

### Sphere formation assay

Cells were plated in 6‐well plates and were cultured in the medium containing B27 (BD Pharmingen, USA), 20 ng/mL basic fibroblast growth factor (bFGF, Invitrogen, USA) and 20 ng/mL epidermal growth factor (EGF, Invitrogen, USA). After 14 days incubation, the cell spheroids were observed under an optical microscope. The experiment was in triplicate.

### Western blot

The proteins were extracted from cells using RIPA lysis buffer (Beyotime, Shanghai, China), followed by quantification using a BCA Protein Assay Kit (Abcam, UK). Then proteins were undergone SDS-PAGE electrophoresis and then transferred to PVDF membranes, followed by sealing with 5% skim milk. Subsequently, the membranes were hatched with primary antibodies at 4 °C overnight and then incubated with horseradish peroxidase-conjugated secondary antibodies. Immunoblots were detected using ECL western blotting substrate (Invitrogen, USA). The experiment was in triplicate.

### Immunofluorescence (IF)

Cells were planted into plates and grown on sterilized coverslips. Then cells were fixed with 4% paraformaldehyde and permeabilized with 0.5% Triton X-100, followed by sealing with 5% defatted milk. Next, the coverslips were incubated with primary antibodies against E-cadherin (Abcam, UK; 1/500) and N-cadherin (Abcam, UK; 1/500) at 4 °C overnight, and then incubated with fluorochrome-labeled secondary antibodies. Finally, the coverslips were stained with DAPI and imaged using a fluorescence microscopy (Nikon, Japan). The experiment went through three independent repeats.

### Fluorescent in situ hybridization (FISH)

GenePharma designed the FISH probes of SOX21-AS1. Cells were fixed and washed, and then were subject to permeabilization with 0.5% Triton X-100. Then, pre-hybridization buffer (Sigma-Aldrich, USA) was added with SOX21-AS1 probe. DAPI solution was used to redye cells and the fluorescent signal was observed under the microscope. The experiment was subject to three independent repeats.

### Subcellular fractionation

PARIS kit was applied to measure the cytoplasmic and nuclear fractions based on instructions. Extracted RNAs were subject to RT‐qPCR analysis to determine the cellular distribution of SOX21-AS1. GAPDH and U6 were served as the cytoplasm control or the nucleus control. The experiment was in triplicate.

### RNA immunoprecipitation (RIP)

RIP assay was performed using the Magna RIP™ RNA-Binding Protein Immunoprecipitation Kit (Millipore, USA) in the light of provider’s descriptions. Cells were lysed in RIP lysis buffer, and immunoprecipitated with antibody against Ago2 (Abcam, UK; 1/50) or USP10 or negative control IgG (CST, USA; 1/20). Precipitated RNA was purified and analyzed by RT-qPCR. The procedure was subject to three independent repeats.

### RNA pull down assay

For RNA–RNA pull down assay, biotinylated miR-576-5p wild-type or mutant probe bought from Sangon Biotech (Shanghai, China) were utilized. Cell lysates was incubated with these biotinylated transcripts for 1 h at 37 °C. The RNA complexes were separated and analyzed by RT-qPCR.

For RNA–protein pull down assay, the biotin-labeled SOX21-AS1 was transcribed in vitro. Cells were mixed with biotinylated SOX21-AS1. Then streptavidin agarose beads (Invitrogen, USA) was added. The associated complex was resolved by SDS-PAGE and went through western blot analysis. The experiment was run in three independent repeats.

### Ubiquitination assay

Ubiquitin, SOX21, and the indicated plasmids were transfected into cells. The lysates were immunoprecipitated with the indicated antibodies on protein A/G beads with rotation. The eluted proteins were detected by western blot. This assay went through three independent repeats.

### Chromatin immunoprecipitation (ChIP)

A ChIP assay kit (Beyotime, Shanghai, China) was used for the ChIP experiment based on the guidance of supplier. Cells were treated with paraformaldehyde for cross-links at room temperature. Cell lysates were then sonicated to get chromatin fragments of 200–300 bp. Then the cell lysates were hatched with anti-STAT6 (Abcam, UK; 1/50) or anti-IgG (CST, USA; 1/20). Precipitated chromatin DNA was purified and then for RT-qPCR analysis. The experiment went through three independent repeats.

### Luciferase reporter assay

pmirGLO dual luciferase vector (Promega, USA) was used to assess the direct binding sites of miR-576-5p on SOX21-AS1 or SOX21 3’UTR. The wild-type or mutant reporter constructs of SOX21-AS1 or SOX21 3’UTR was co-transfected with miR-576-5p mimics or NC mimics into cells for 48 transfection. The relative luciferase activity was measured using a Dual-Luciferase Reporter Assay kit (Promega, USA) and normalized to Renilla luciferase activity. The experiment was in triplicate.

### Xenograft tumor model

The nude mice (4–6-week old) were purchased and maintained at the Experimental Animal Center of Shanghai Laboratory Animal Center, Chinese Academy of Sciences in SPF barrier facilities. PC cells stably transfected with sh-SOX21-AS1#1#1 or shRNA were re-suspended at 1 × 10^8^ cells/ml. For subcutaneous tumorigenicity, a total of 100 μl of suspended cells were subcutaneously injected into the right bilateral hind legs of mice. The size of tumor volume was calculated every four days. Animals were sacrificed by cervical dislocation at 28 days post injection. The tumors were collected for further study. IHC was taken to detect the expression of E-cadherin, N-cadherin, Ki67 and PCNA of the tumor xenografts tissue to evaluate the proliferation area. The animal experiments were approved by the Animal Care Committee of Shanghai Municipal Hospital of Traditional Chinese Medicine, Shanghai University of Traditional Chinese Medicine (ethical code: T-No202204200S0720102 [202]).

### Database application

This study was conducted with the application of many databases. GEPIA2 (http://gepia2.cancer-pku.cn/) database was applied to analyze the correlation of SOX21-AS1 expression and the overall survival of patients with PC. It was also utilized when we searched the co-expressed gene with SOX21-AS1 in PC. SOX21-AS1 expression in PC tissues was exhibited through TCGA database. UCSC (http://genome.ucsc.edu/) database was applied to confirm whether SOX21 was the nearby gene of SOX21-AS1. We applied starBase (http://starbase.sysu.edu.cn) to search potential miRNAs of SOX21-AS1 and SOX21. AnnoLnc (http://annolnc.gao-lab.org/) and JASPAR (http://jaspar.genereg.net/) databases were utilized to predict potential transcription factors combined with SOX21-AS1 promoter.

### Statistical analysis

The experiments were undertaken independently for three times. Statistical analysis was analyzed using SPSS software. The analysis of data was performed using Student’s *t*-test and ANOVA between different groups. Experimental resells were exhibited as means ± SD. p value below 0.05 was referred as data with statistical significance.

## Results

### SOX21-AS1 silence suppressed PC progression

**Fig. 1:**
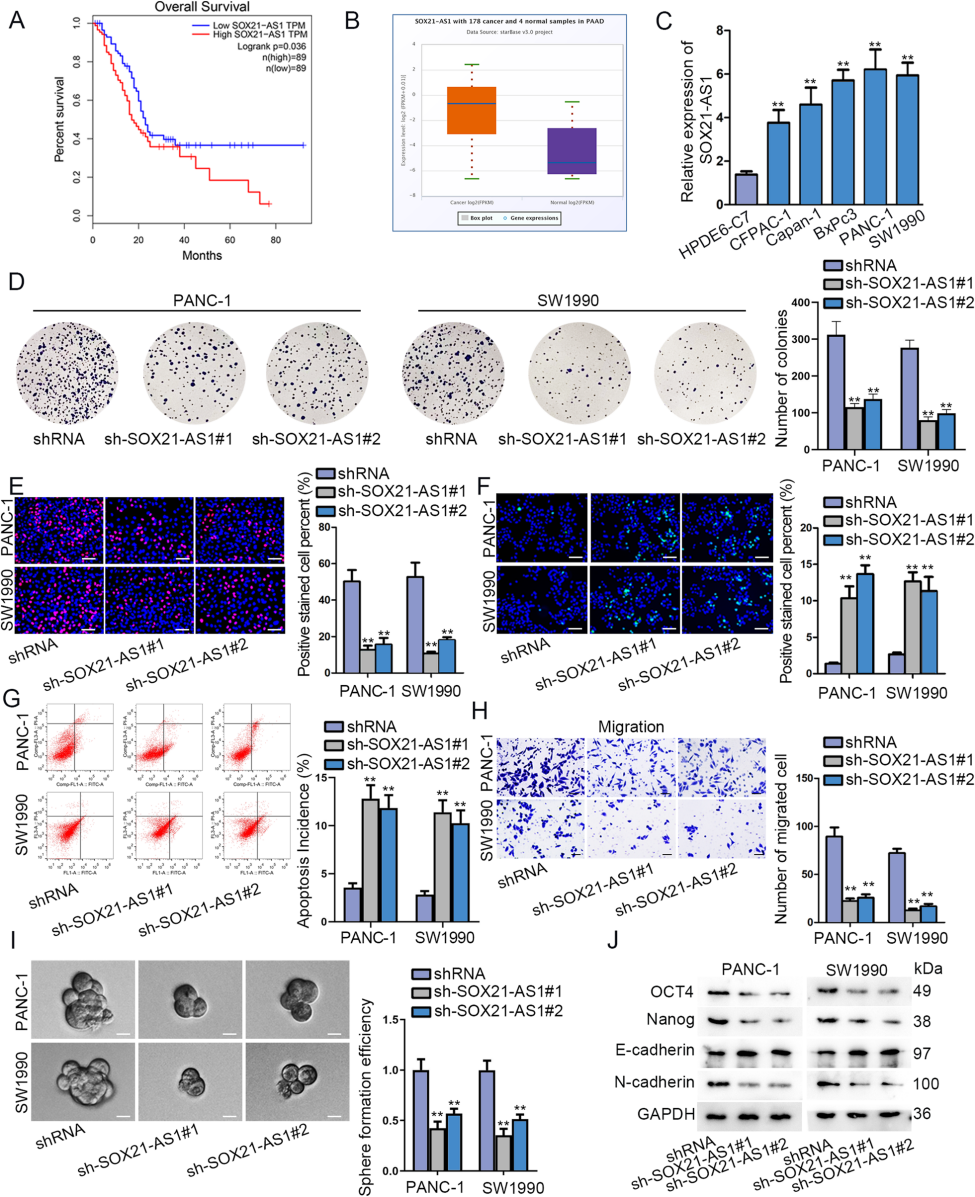
SOX21-AS1 silence suppressed PC progression.** A** GEPIA2 analyzed the relation between SOX21-AS1 expression and the survival of PC patients. **B** TCGA database predicted SOX21-AS1 expression in PC tissues. **C** SOX21-AS1 expression in PC cells. **D**, **E** PC cell proliferation after SOX21-AS1 silence. **F**, **G** TUNEL assay as well as flow cytometry detected the apoptosis process in SOX21-AS1 silenced PC cells. **H** Transwell assays detected the migration property in SOX21-AS1 silenced PC cells. **I** Sphere formation assays detected the stemness in SOX21-AS1 silenced PC cells. **J** The protein levels of EMT markers and transcription factors in SOX21-AS1 silenced PC cells. ^**^P < 0.01

At first, we applied GEPIA2 (http://gepia2.cancer-pku.cn/) database to analyze the correlation of SOX21-AS1 expression and the overall survival of patients with PC. As indicated in Fig. 1A, patients with high expression of SOX21-AS1 were accompanied with short survival time (p = 0.036, n = 89). Accordantly, data from TCGA database displayed a higher SOX21-AS1 expression in PC tissues than normal tissues (Fig. 1B). Consistently, PC cells including CFPAC-1, Capan-1, BxPc3, PANC-1 and SW1990 harbored high expression of SOX21-AS1 compared to human normal pancreatic duct epithelial cell HPDE6-C7 (Fig. 1C). We next transfected two specific shRNAs into two PC cells PANC-1 and SW1990 to silence SOX21-AS1 expression, and then conducted loss-of function experiments (Additional file 1: Figure S1A). As revealed in colony formation and EdU assays, SOX21-AS1 knockdown reduced the proliferative ability in PC cells (Fig. 1D-E). Besides, the apoptosis rate was elevated in SOX21-AS1 silenced PC cells, as manifested by TUNEL and flow cytometry assays (Fig. 1F-G). In parallel, SOX21-AS1 deletion also repressed the number of migrated PC cells (Fig. 1H). It was uncovered in sphere formation assays that SOX21-AS1 silence obviously inhibited the sphere formation efficiency of PC cells (Fig. 1I). Through IF assays, we found that the intensity of E-cadherin was strengthened after SOX21-AS1 interference, while N-cadherin displayed declined intensity (Additional file 1: Figure S1B). Additionally, western blot data showed that after SOX21-AS1 reduction, E-cadherin protein expression increased, while OCT4, Nanog and N-cadherin exhibited reduced expression (Fig. 1J). Same results were obtained from CFPAC-1 and BxPc3, another two PC cell lines (Additional file 2: Figure S2A-G). The above findings all demonstrated the oncogenic property of SOX21-AS1 in the regulation of PC cell malignancy.

### SOX21-AS1 regulated its nearby gene SOX21 at a post-transcriptional level

**Fig. 2:**
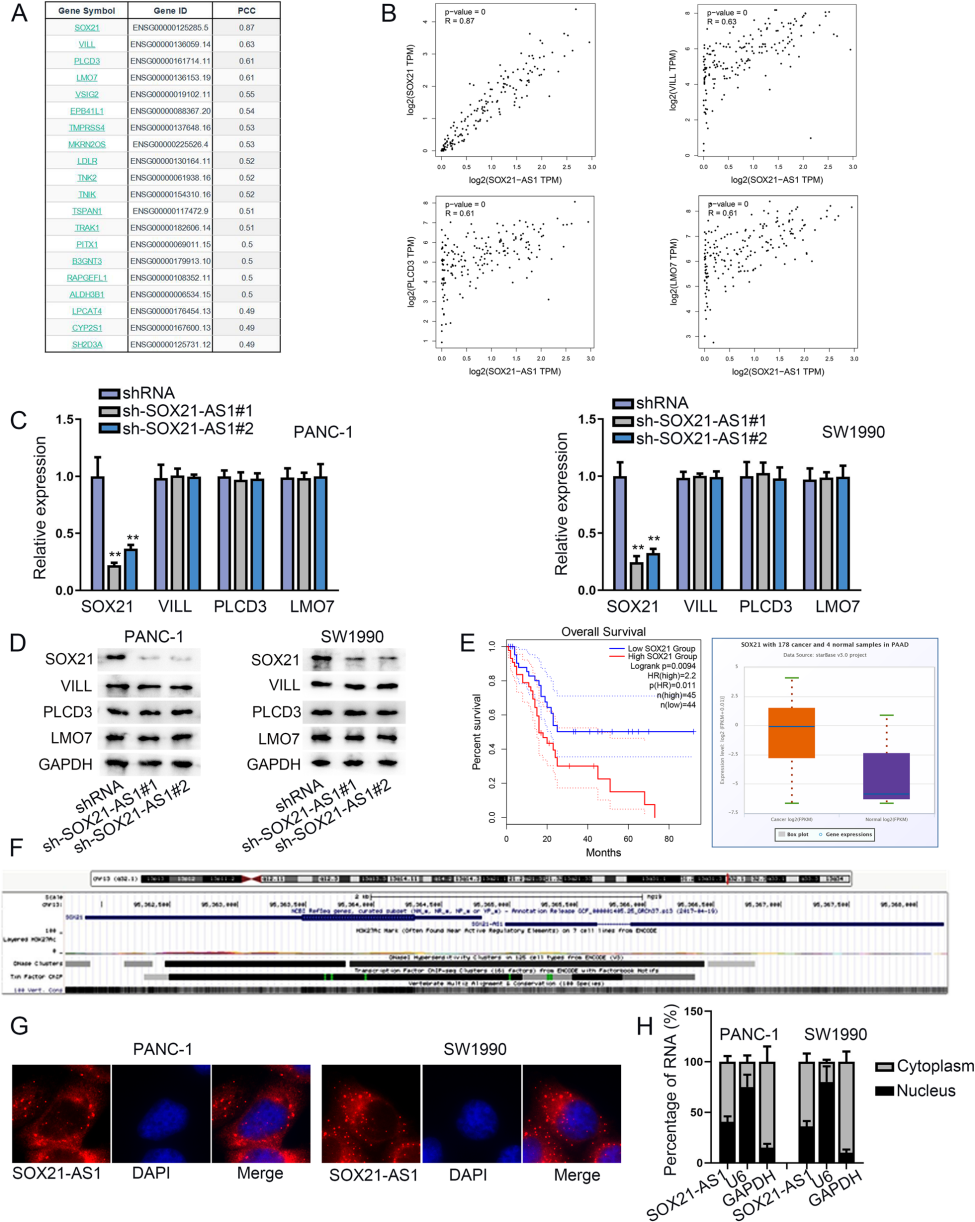
SOX21-AS1 regulated its nearby gene SOX21 at a post-transcriptional level.** A** GEPIA2 displayed the co-expressed gene with SOX21-AS1. **B** GEPIA2 displayed the correlation between SOX21-AS1 and SOX21, VILL, PLCD3 or LMO7 in PC. **C**, **D** The transfection efficiency of shRNAs targeting these four genes.** E** GEPIA 2 database displayed the relation between SOX21 expression and PC patients’ survival time. TCGA database displayed the expression of SOX21 in PC tissues. **F** UCSC database disclosed the location of SOX21-AS1 and SOX21 on the chromatin. **G**, **H** The cellular location of SOX21-AS1 in PC cells confirmed via FISH assay along with subcellular fractionation detection. ^**^P < 0.01

We next searched the co-expressed gene with SOX21-AS1 via GEPIA2, and screened the top 4 genes (SOX21, VILL, PLCD3 and LMO7) for further screening (Fig. 2A). The data from GEPIA2 exhibited the positive correlation between SOX21-AS1 and these four genes in PC tissues (Fig. 2B). We further discovered that SOX21-AS1 silence declined the mRNA and protein levels of SOX21, whereas had no changes on those of the other genes (Fig. 2C, D). Subsequently, it was found that PC patients with high SOX21 expression had short survival time, and SOX21 displayed high expression in PC tissues (Fig. 2E). Furthermore, we confirmed that SOX21 was the nearby gene of SOX21-AS1 through UCSC (http://genome.ucsc.edu/) database (Fig. 2F). LncRNAs exerting their functions by cooperating with their nearby genes has been testified [20]. Thereby, to further confirm the regulatory model of which SOX21-AS1 on SOX21 in PC cells, we confirmed the cellular location of SOX21-AS1 in PC cells via FISH assay along with subcellular fractionation analysis, which confirmed the main location of SOX21-AS1 in the cytoplasm, suggesting the possibility of SOX21-AS1 regulating SOX21 at a post-transcriptional level (Fig. 2G, H). Collectively, SOX21-AS1 regulated its nearby gene SOX21 at a post-transcriptional level.

We further investigated the interaction between SOX21-AS1 and SOX21 on PC progression, we up-regulated the level of SOX21 in PC cells (Additional file 1: Figure S1C), and found that SOX21 overexpression reversed the inhibited proliferation property in SOX21-AS1 silenced cells (Additional file 3: Figure S3A, B). The enhanced cell apoptosis led by SOX21-AS1 deletion was counteracted by co-transfection of pcDNA-SOX21 (Additional file 3: Figure S3C). Moreover, the suppressed migration capacity in SOX21-AS1 silenced cells was restored by co-transfection of pcDNA-SOX21 (Additional file 3: Figure S3D). The results from sphere formation assays indicated that SOX21 increase overturned the impaired effects of SOX21-AS1 knockdown on the stemness (Additional file 3: Figure S3E). Simultaneously, SOX21 overexpression could counteract the inhibited EMT process after SOX21-AS1 interference (Additional file 1: Figure S3F).

### SOX21-AS1 acted as a ceRNA to target miR-576-5p/SOX21 axis

**Fig. 3:**
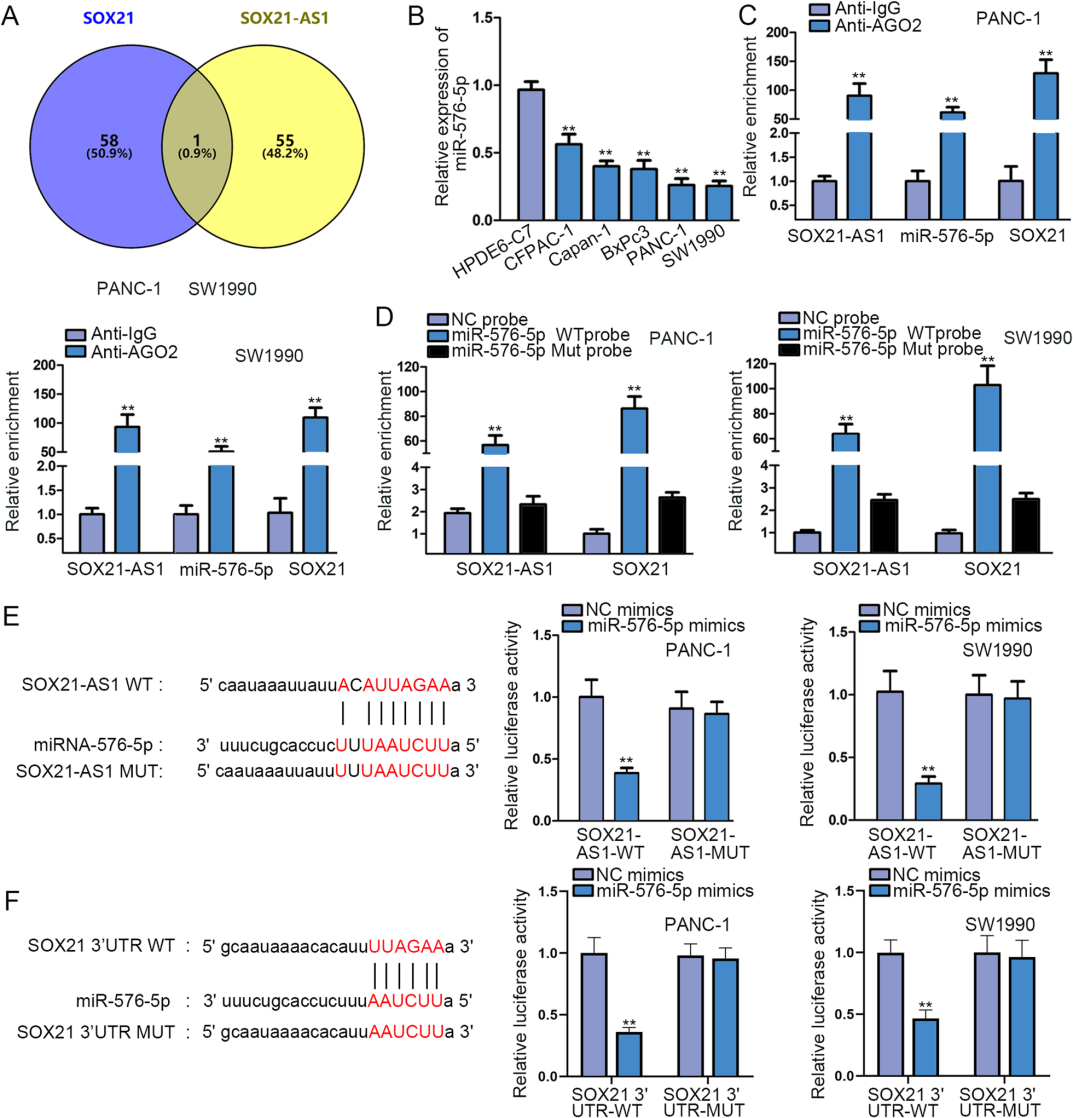
SOX21-AS1 acted as a ceRNA to target miR-576-5p/SOX21 axis.** A** Venn diagram revealed the common miRNA combined with SOX21-AS1 and SOX21. **B** RT-qPCR analyzed miR-576-5p expression in PC cells. **C** SOX21-AS1, miR-576-5p and SOX21 enrichment was measured in Ago2 groups and negative control groups. **D** RNA pull down assays detected the enrichment of SOX21-AS1 and SOX21 in miR-576-5p WT probe and miR-576-5p Mut probe groups. **E**, **F** StarBase database, along with luciferase reporter assays verified the binding of miR-576-5p on SOX21-AS1 and SOX21. ^**^P < 0.01

Through starBase (http://starbase.sysu.edu.cn), potential miRNAs that may combine with SOX21-AS1 and SOX21 were predicted. The results from Venn diagram displayed that only one miRNA (miR-576-5p) met the requirement (Fig. 3A). The low expression of miR-576-5p was verified in PC cells (Fig. 3B). The data from RIP assays showed that SOX21-AS1, miR-576-5p and SOX21 co-existence in the RNA-induced silencing complex (RISC), as demonstrated by the highly enrichment of these three RNAs in Ago2 groups (Fig. 3C). Meanwhile, we confirmed that SOX21-AS1 and SOX21 were both largely enriched in miR-576-5p WT probe groups, while had no changes in miR-576-5p MUT probe groups (Fig. 3D). Additionally, we separately predicted the binding sites of miR-576-5p on SOX21-AS1 and SOX21, and then we validated that miR-576-5p overexpression significantly reduced the luciferase activity of SOX21-AS1-WT and SOX21-WT, while the corresponding mutant groups displayed no difference (Additional file 1: Figure S1D and Fig. 3E, F).

Furthermore, we carried out rescue experiments to explore whether SOX21-AS1 may regulate SOX21 expression and PC progression via sponging miR-576-5p. It was found that SOX21 expression and protein was reduced by SOX21-AS1 depletion, but this effect was partially offset by silencing miR-576-5p expression (Additional file 4: Figure S4A, B). Besides, we found that miR-576-5p down-regulation partly recovered the lessened proliferation ability caused by SOX21-AS1 silence (Additional file 4: Figure S4C, D). It was unveiled in TUNEL assays that miR-576-5p silence could counteract the elevated apoptosis in SOX21-AS1 silenced cells (Additional file 4: Figure S4E). Moreover, miR-576-5p inhibition could partly restore the repressed migration ability and stemness in SOX21-AS1 down-regulated PANC-1 and SW1990 cells (Additional file 4: Figure S4F, G). Additionally, the lessened EMT process caused by SOX21-AS1 deletion was partly rescued by miR-576-5p repression together (Additional file 4: Figure S4H).

### SOX21-AS1 interacted with USP10 to deubiquitinate and stabilized SOX21 protein

**Fig. 4:**
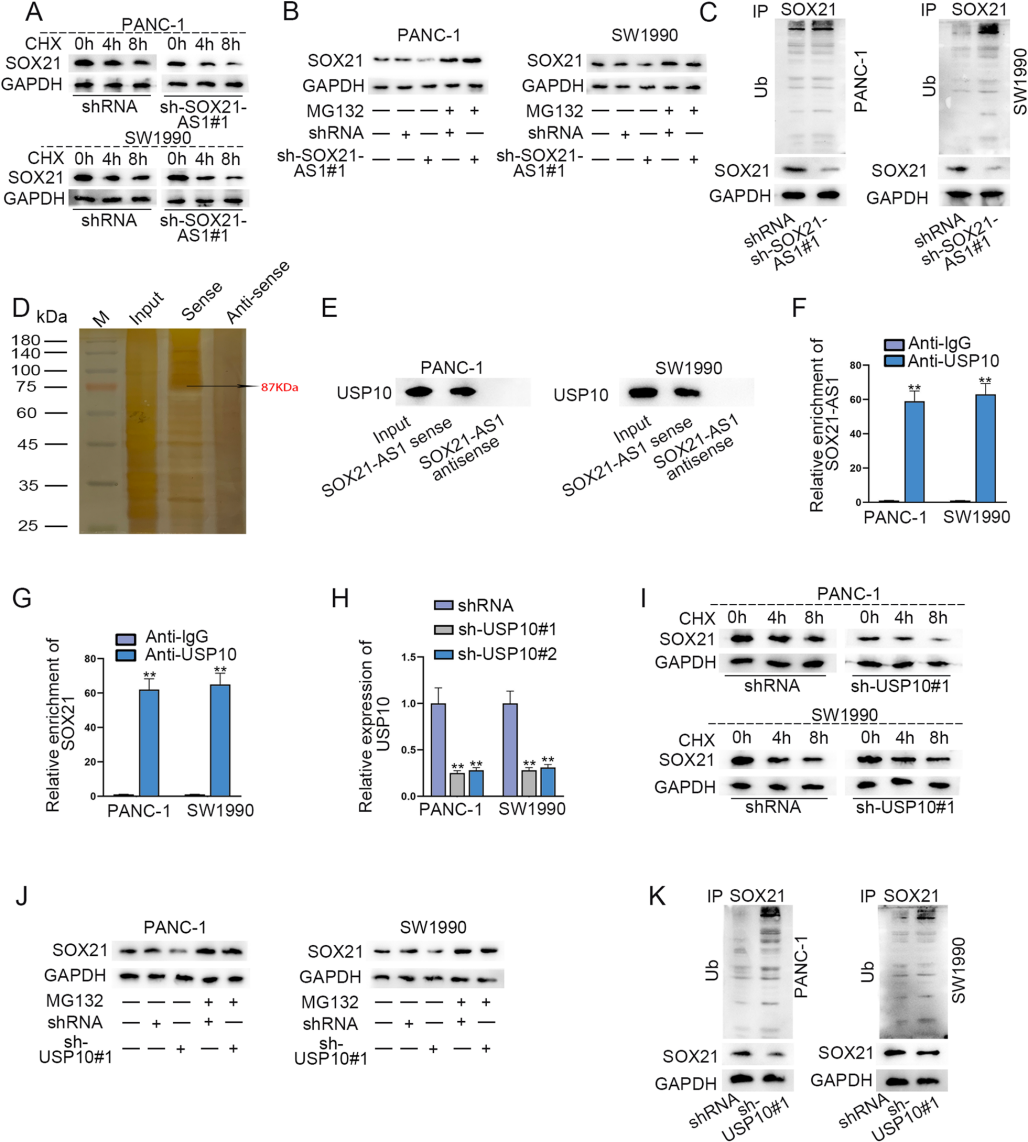
SOX21-AS1 interacted with USP10 to deubiquitinate and stabilized SOX21 protein. **A** SOX21 protein level in sh-SOX21-AS1 transfected PC cells upon CHX treatment. **B** SOX21 protein level in sh-SOX21-AS1 transfected PC cells upon MG132 treatment. **C** Ubiquitination of SOX21 protein in two PC cells transfected with SOX21-AS1-shRNA. **D** Biotinylated sense and antisense SOX21-AS1 were transcribed in vitro a for RNA pull down assays. A specific band at about 87 kDa was excised after sliver staining. **E** Immunoblotting indicated specific association of USP10 and SOX21-AS1. **F**, **G** RIP assays confirmed the combination between USP10 and SOX21-AS1/SOX21. **H** Down-regulation efficiency of USP10 in two PC cells was verified via RT-aPCR and western blot. **I** SOX21 protein level in sh-USP10 transfected PC cells upon CHX treatment. **J** SOX21 protein level in sh-USP10 transfected PC cells upon MG132 treatment. **K** Ubiquitination of SOX21 protein in two PC cells transfected with USP10-shRNA. ^**^P < 0.01

We further treated two PC cells with the protein synthesis inhibitor CHX and measured the stability of the SOX21 protein. The results showed that the stability of SOX21 protein was decreased when SOX21-AS1 was down-regulated (Fig. 4A), and this effect was attenuated after treatment with proteasome inhibitor MG132 (Fig. 4B). Moreover, the ubiquitination of SOX21 protein was increased after SOX21-AS1 depletion (Fig. 4C). To uncover how SOX21-AS1 regulates the stability of SOX21 protein, we tried to identify the protein partners of SOX21-AS1 in PC cells using RNA pull down assay. One specific band exhibited on the electrophoretic gel at approximately 87 kDa in contrast to the antisense SOX21-AS1 (Fig. 4D). Then the gel was subjected to mass spectrometry and we finally identified SOX21-AS1-interacting protein USP10. Western blot and RIP analyses further confirmed the combination between SOX21-AS1 and USP10 (Fig. 4E, F). Also, the binding of SOX21 and USP10 was also verified by RIP assay (Fig. 4G). USP10 is a cytoplasmic ubiquitin-specific protease which can deubiquitinate and stabilize protein [21]. Thus we further supposed that SOX21-AS1 might interact with USP10 to deubiquitinate and stabilize SOX21 protein. To prove our assumption, we silenced USP10 expression and treated USP10-shRNA into two PC cells together with CHX to measure USP10 protein level. We could see that USP10 down-regulation inhibited the half-life of SOX21 protein (Fig. 4H, I), but this phenomenon was reversed after MG132 treatment (Fig. 4J). As expected, the ubiquitination of SOX21 protein was enhanced when USP10 expression was reduced (Fig. 4K).

### STAT6 transcriptionally activated the expression of SOX21-AS1 and SOX21

**Fig. 5:**
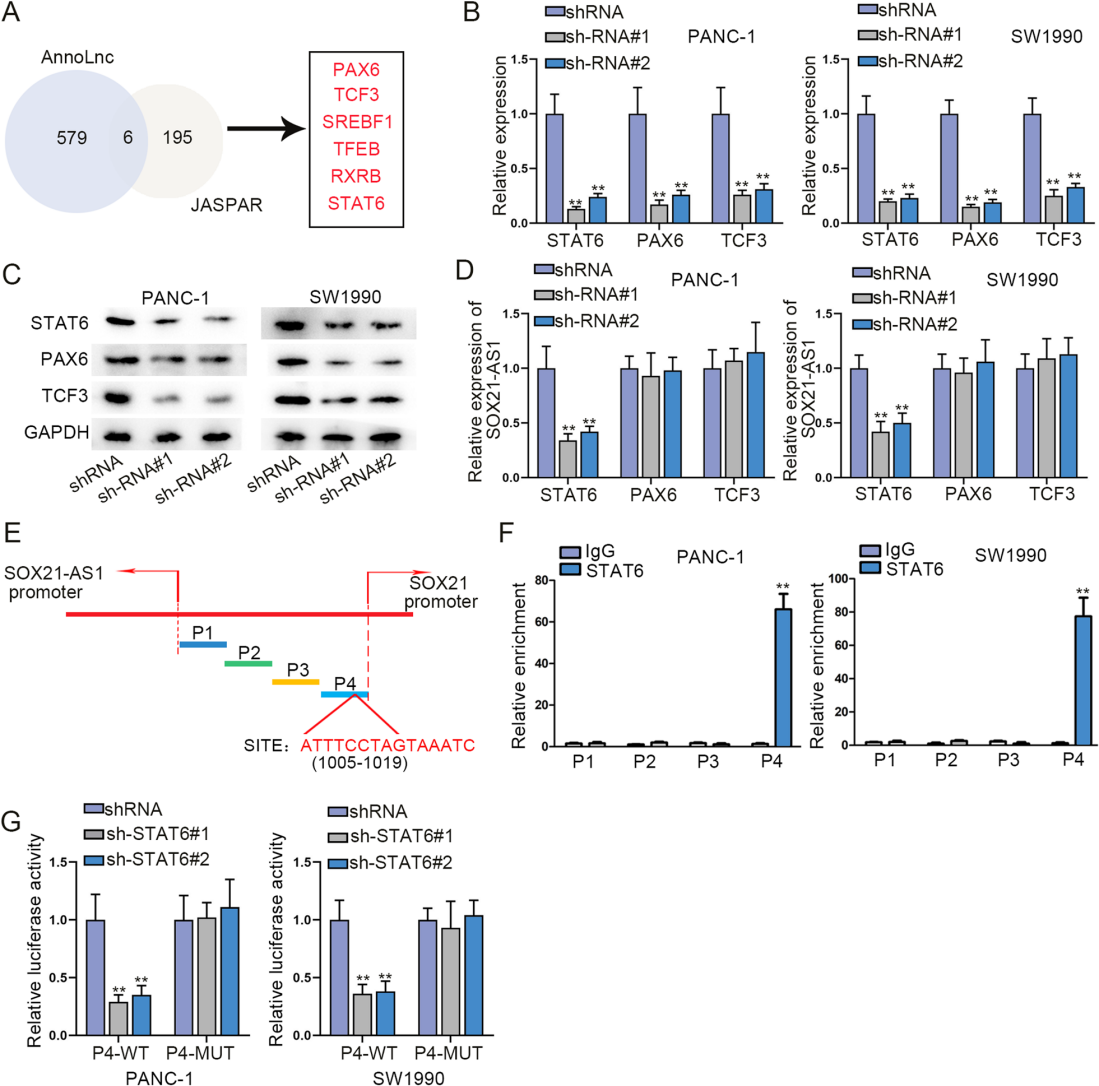
STAT6 transcriptionally activated the expression of SOX21-AS1 and SOX21.** A** AnnoLnc and JASPAR database predicted potential six transcription factors combined with SOX21-AS1 promoter. **B**, **C** The transfection efficiency of shRNAs targeting three transcription factors (STAT6, PAX6 and TCF3). **D** SOX21-AS1 expression in PC cells transfected with shRNAs targeting three transcription factors. **E** The binding sites of STAT6 on SOX21-AS1 promoter and SOX21 promoter. **F** ChIP data of the combining capabilities between P1, P2, P3 and P4 sites and STAT6. **G** The luciferase activity of P4-WT and P4-MUT in PANC-1 and SW1990 cells transfected with shRNAs targeting STAT6. ^**^P < 0.01

Through AnnoLnc (http://annolnc.gao-lab.org/) and JASPAR (http://jaspar.genereg.net/) database, we predicted potential six transcription factors combined with SOX21-AS1 promoter (Fig. 5A). SOX21-AS1 expression was significantly declined when STAT6 was silenced, while the other candidates showed no variation (Fig. 5B–D), so STAT6 was chosen for further analyses. We found that STAT6 combined with SOX21-AS1 promoter and SOX21 promoter at four sites (Fig. 5E), and ChIP assays further validated that both SOX21-AS1 promoter and SOX21 promoter were enriched in STAT6 precipitates at P4 sites (Fig. 5F). Additionally, we found that P4-WT group displayed reduced luciferase activity after STAT6 silence, while the corresponding mutant group was barely affected (Fig. 5G). Conclusively, STAT6 transcriptionally activated the expression of SOX21-AS1 and SOX21.

### SOX21-AS1 silence inhibited tumor growth in PC

**Fig. 6:**
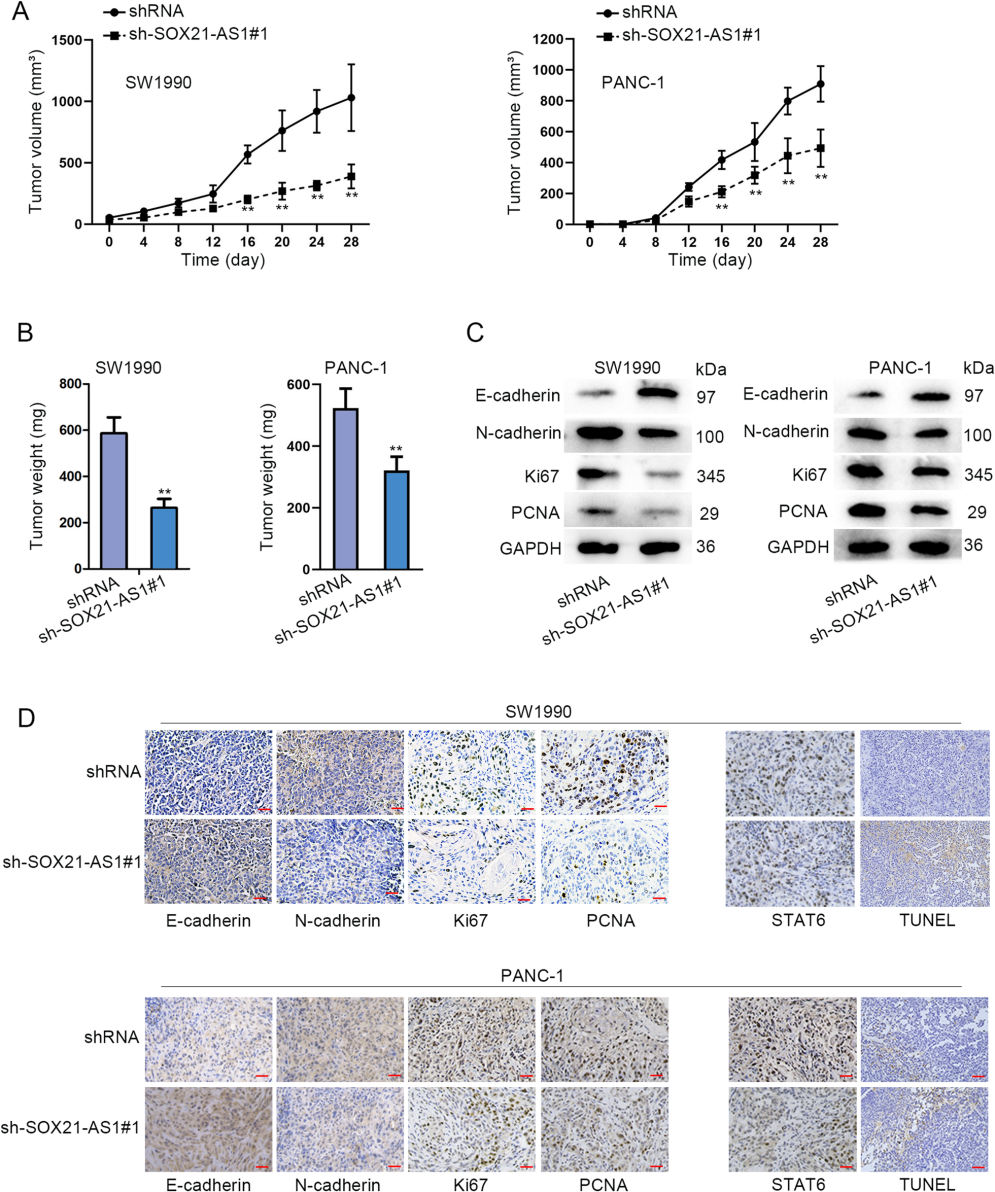
SOX21-AS1 silence inhibited tumor growth in PC. **A**, **B** Tumor volume along with tumor weight upon SOX21-AS1 silence. **C**, **D** Western blot and immunohistochemistry analyses detected the EMT process when SOX21-AS1 was silenced, along with the apoptosis of tumor and STAT6 expression. ^**^P < 0.01

In addition, we also performed in vivo experiments by establishing a xenograft tumor model to verify the impacts SOX21-AS1 may exert on tumor growth. According to the result, the sh-SOX21-AS1#1 group revealed an obviously lower speed of tumor volume and tumor weight compared with the empty vector group (Fig. 6A, B). Moreover, we found that SOX21-AS1 silence inhibited the EMT process according to western blot and immunohistochemistry analyses (Fig. 6C, D). Moreover, it was shown that after SOX21-AS1 silence, the apoptosis of tumor enhanced, while the expression of STAT6 exhibited no obvious change between different groups. Taken together, SOX21-AS1 silence inhibited tumor growth in PC.

## Discussion

**Fig. 7:**
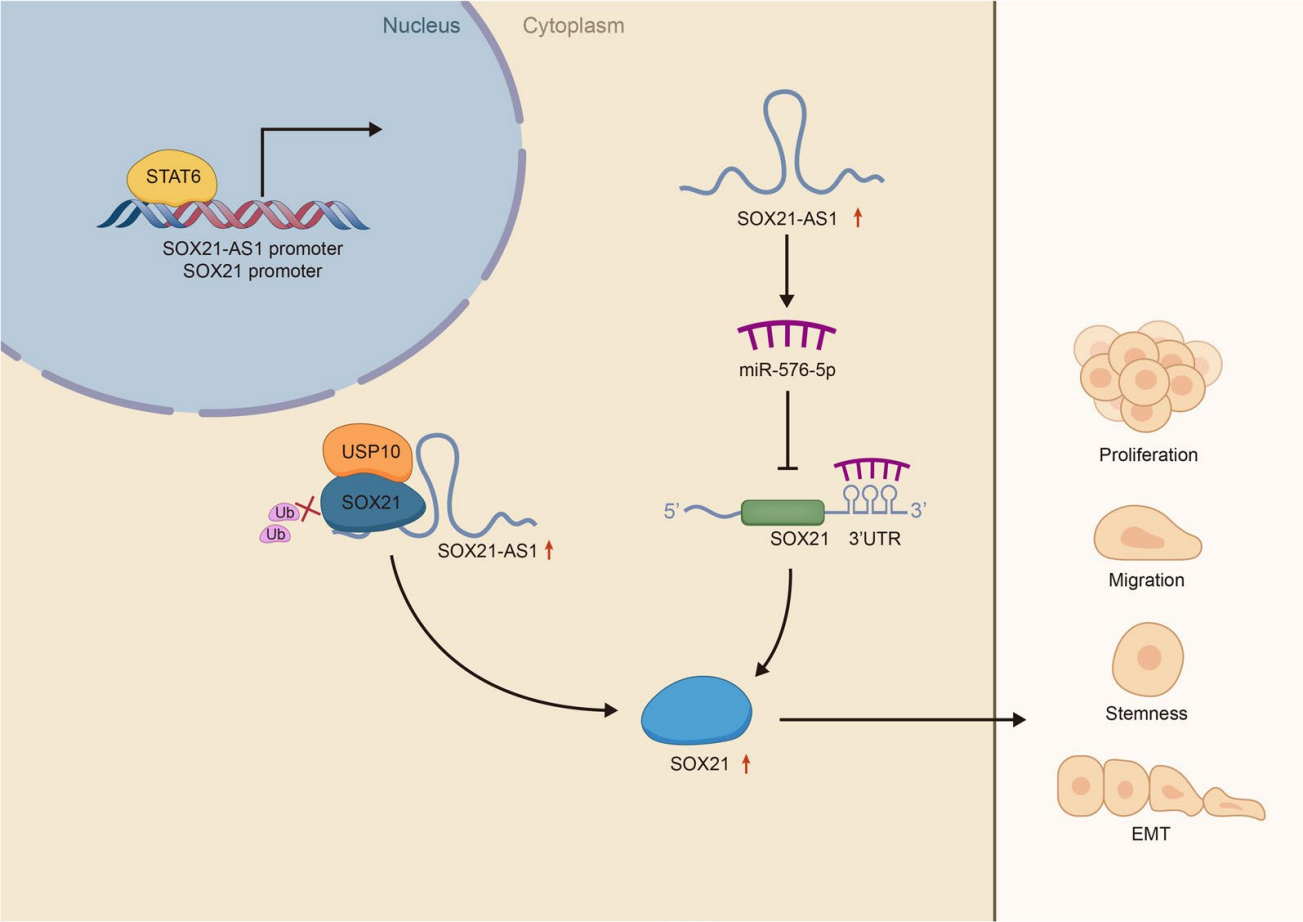
Concept map illustrated the regulatory mechanism of SOX21-AS1 on regulating PC progression

In our study, we elucidated a new putative mechanism by which STAT6 transcriptionally activated SOX21-AS1 regulated its nearby gene SOX21 via acting as a ceRNA to target miR-576-5p and interacting with USP10 in a manner important for PC cell proliferation, apoptosis, migration and EMT (Fig. 7).

In recent years, a large number of reports have demonstrated the close relationship lncRNAs possess with the tumorigenesis of PC [22]. The focus of our study, SOX21-AS1, is a relatively novel lncRNA. It has been elucidated in many cancers, such as oral cancer [23], hepatocellular carcinoma [24], lung adenocarcinoma [25], nephroblastoma [26] and osteosarcoma [27] in which SOX21-AS1 expression was testified to be higher in cancer cells. Consistent with these findings, we revealed the high expression pattern of SOX21-AS1 in PC cells, and SOX21-AS1 deletion obviously repressed PC progression in vitro and tumor growth in vivo. It was the first time that we had verified SOX21-AS1 as a potential regulatory molecule in the regulation of PC cells.

LncRNAs have been described to interact with their nearby genes in the modulation of cancer cells [28, 29]. In our research, SOX21 was verified to be the nearby gene of SOX21-AS1, and it was positively regulated by SOX21-AS1 in PC cells. As reported previously, overexpression of SOX21 induces glioma cell apoptosis [30]. SOX21 promoter is a candidate noninvasive diagnostic biomarker for colorectal cancer [31]. Our study also proved that SOX21 was highly expressed in PC, and rescue experiments further validated that SOX21-AS1 aggravated PC cell malignancy via enhancing SOX21 expression.

Cytoplasmic lncRNAs have emerged as ceRNAs in cancer development, including PC [32, 33]. Through bioinformatics analysis and related mechanism assays, miR-576-5p was proven to be the target miRNA of SOX21-AS1, and the ceRNA model was then uncovered in PC. MiR-576-5p has been documented to increase the cell migration and invasion in esophageal squamous cell carcinoma [34]. MiR-576-5p has been documented to aggravate colorectal cancer cell malignancy [35, 36]. Besides, miR-576-5p has been reported to be sponged by linc-PINT in esophageal cancer [37]. In line with these research outcomes, we verified the low miR-576-5p expression in PC cells.

Since the experimental result of rescue assays in our study showed that miR-576-5p interference only partially offset the suppression on PC cell behaviors caused by SOX21-AS1 knockdown, we predicted that SOX21-AS1 may regulate PC cells via another pathway. Through mechanism experiments, we found that SOX21-AS1 could recruit USP10 to deubiquitinate and stabilize SOX21 protein. Furthermore, the ubiquitination of SOX21 protein was enhanced after USP10 expression was reduced in PC cells. USP10 is a member of the deubiquitinases (DUBs), and many studies have uncovered that USP10 can regulate protein stability by deubiquitination [38, 39]. USP10 has also been found to be targeted by miR-191 and thus contributing to the inhibition of PC [40]. What we revealed about USP10 on regulating SOX21 protein may help to provide some theoretical guidance for OC treatment in the future.

At last, it was verified that STAT6 may be responsible for the up-regulation of SOX21-AS1 in PC as it could transcriptionally activate SOX21-AS1 and SOX21 expression in PC cells. STAT6 promotes the proliferation of colorectal cancer and breast cancer cells [41], but how STAT6 may exert certain functions on the biological properties of PC cells may need further exploration.

## Conclusion

Our study elucidated that SOX21-AS1 played a tumor promoting role in PC, and a mechanism was further revealed whereby STAT6-activated SOX21-AS1 promoted PC cell malignancy via up-regulation of SOX21. Utilization of these results in clinical practice may contribute to the diagnosis and treatment for PC patients.

## Supplementary Information

**Additional file 1: Figure S1.** Transfection efficiency of RNAs. **A.** SOX21-AS1 expression was reduced in PC cells via transfecting shRNAs targeting SOX21-AS1. **B** IF assays detected the intensity of EMT markers in sh-SOX21-AS1 transfected PC cells. **C** SOX21 expression was elevated in PC cells by pcDNA-SOX21 transfection. **D** MiR-576-5p expression was elevated by miR-576-5p mimics transfection. ^**^P < 0.01.

**Additional file 2: Figure S2.** SOX21-AS1 silence suppressed the progression of PC. **A-G** Loss-of-function assays were performed in another two PC cell lines (CFPAC-1 and BxPc3) to further verify the malignant cell behaviors including proliferation, migration, EMT as well as apoptosis upon SOX21 silence treatment. ^**^P < 0.01.

**Additional file 3: Figure S3.** SOX21-AS1 affected PC cell proliferation, apoptosis, migration, stemness and EMT via modulating SOX21 expression. Rescue experiments in PC cells transfected with shRNA, sh-SOX21-AS1#1 and sh-SOX21-AS1#1 + pcDNA-SOX21, respectively. **A-B** Cell proliferation detection. **C** Cell apoptosis detection. **D** Transwell assays detected the migration ability. **E** Sphere formation assays detected the stemness.** F** Western blot analyzed the protein levels of EMT markers and transcription factors. ^**^P < 0.01.

**Additional file 4: Figure S4.** SOX21-AS1 affected PC cell proliferation, apoptosis, migration, stemness and EMT via interacting with miR-576-5p.** A** MiR-576-5p expression was decreased in PC cells. Rescue experiments were conducted in PC cells transfected with shRNA, sh-SOX21-AS1#1 and sh-SOX21-AS1#1 + miR-576-5p inhibitor, respectively. **B** SOX21 mRNA along with protein levels. **C**, **D** Cell proliferation detection. **E** TUNEL assays detected the cell apoptosis. **F** The migration of PC cells was testified through Transwell assays. **G** Sphere formation assays detected the stemness.** H** Western blot analyzed the protein levels of EMT markers and transcription factors. ^*^P < 0.05, ^**^P < 0.01

## Data Availability

Not applicable.

## Abbreviations

PC: Pancreatic cancer
lncRNAs: Long non-coding RNAs
EMT: Epithelial-mesenchymal transition
SOX21: SRY-box transcription factor 21
USP10: Ubiquitin-specific peptidase 10
STAT6: Signal transducer and activator of transcription 6
ncRNAs: Non-coding RNAs
ceRNAs: Competing endogenous RNAs
miRNAs: MicroRNAs
SOX21-AS1: SRY-box transcription factor 21 antisense divergent transcript 1
RT-qPCR: Quantitative real-time polymerase chain reaction
EdU: 5-Ethynyl-2’-deoxyuridine
TUNEL: Terminal-deoxynucleoitidyl Transferase Mediated Nick End labeling
IF: Immunofluorescence
FISH: Fluorescent in situ hybridization
RIP: RNA immunoprecipitation
ChIP: Chromatin immunoprecipitation
