# Supplementary figures and images for: SOX21-AS1 activated by STAT6 promotes pancreatic cancer progression via up-regulation of SOX21
Source: J Transl Med. 2022 Nov 5;20:511. doi: 10.1186/s12967-022-03521-5 (PMC9636668; doi:10.1186/s12967-022-03521-5)

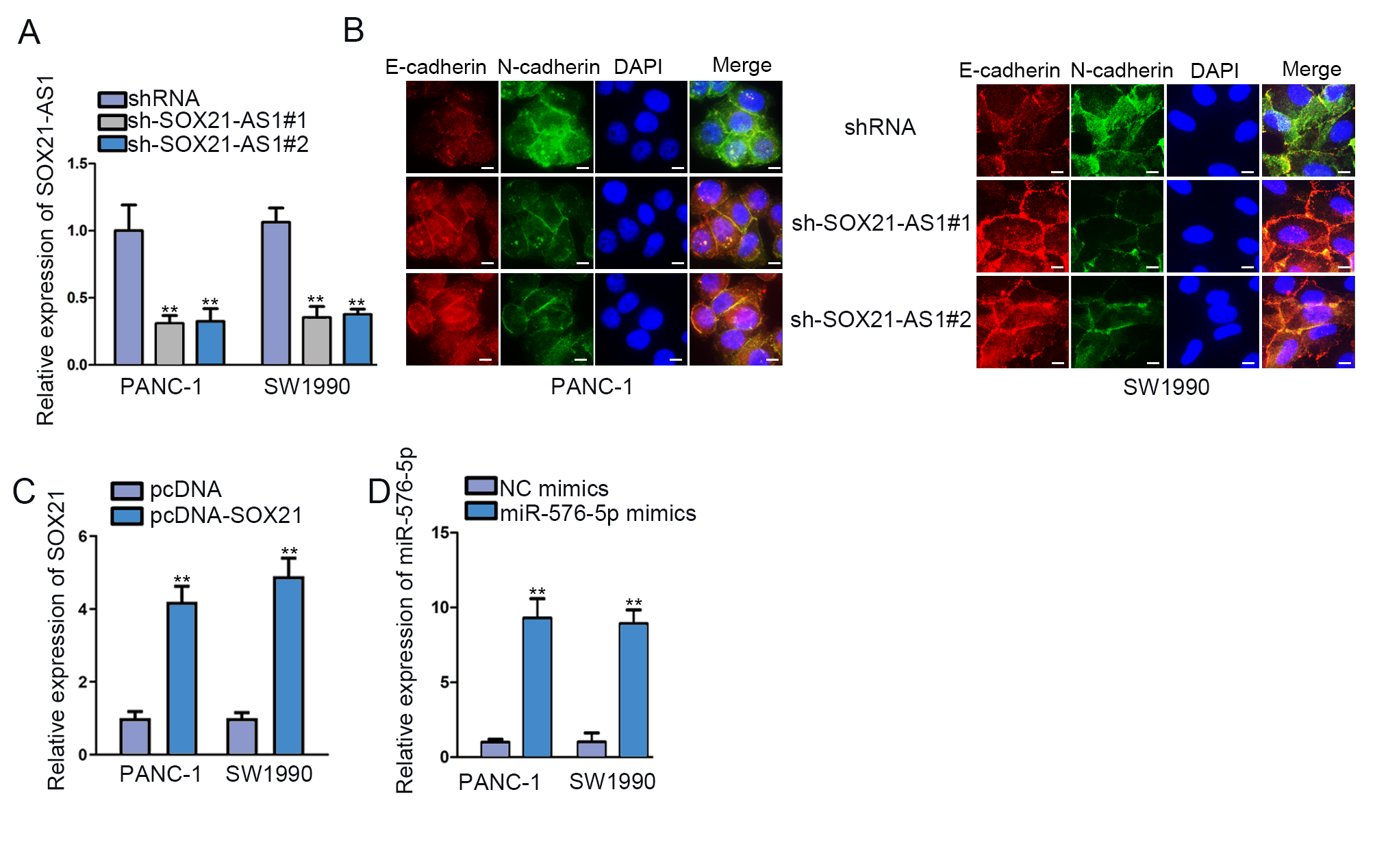

Supplement: Supplementary file 1 — Additional file 1: Figure S1. Transfection efficiency of RNAs. A. SOX21-AS1 expression was reduced in PC cells via transfecting shRNAs targeting SOX21-AS1. B IF assays detected the intensity of EMT markers in sh-SOX21-AS1 transfected PC cells. C SOX21 expression was elevated in PC cells by pcDNA-SOX21 transfection. D MiR-576-5p expression was elevated by miR-576-5p mimics transfection. **P < 0.01. [file 12967_2022_3521_MOESM1_ESM.tif]

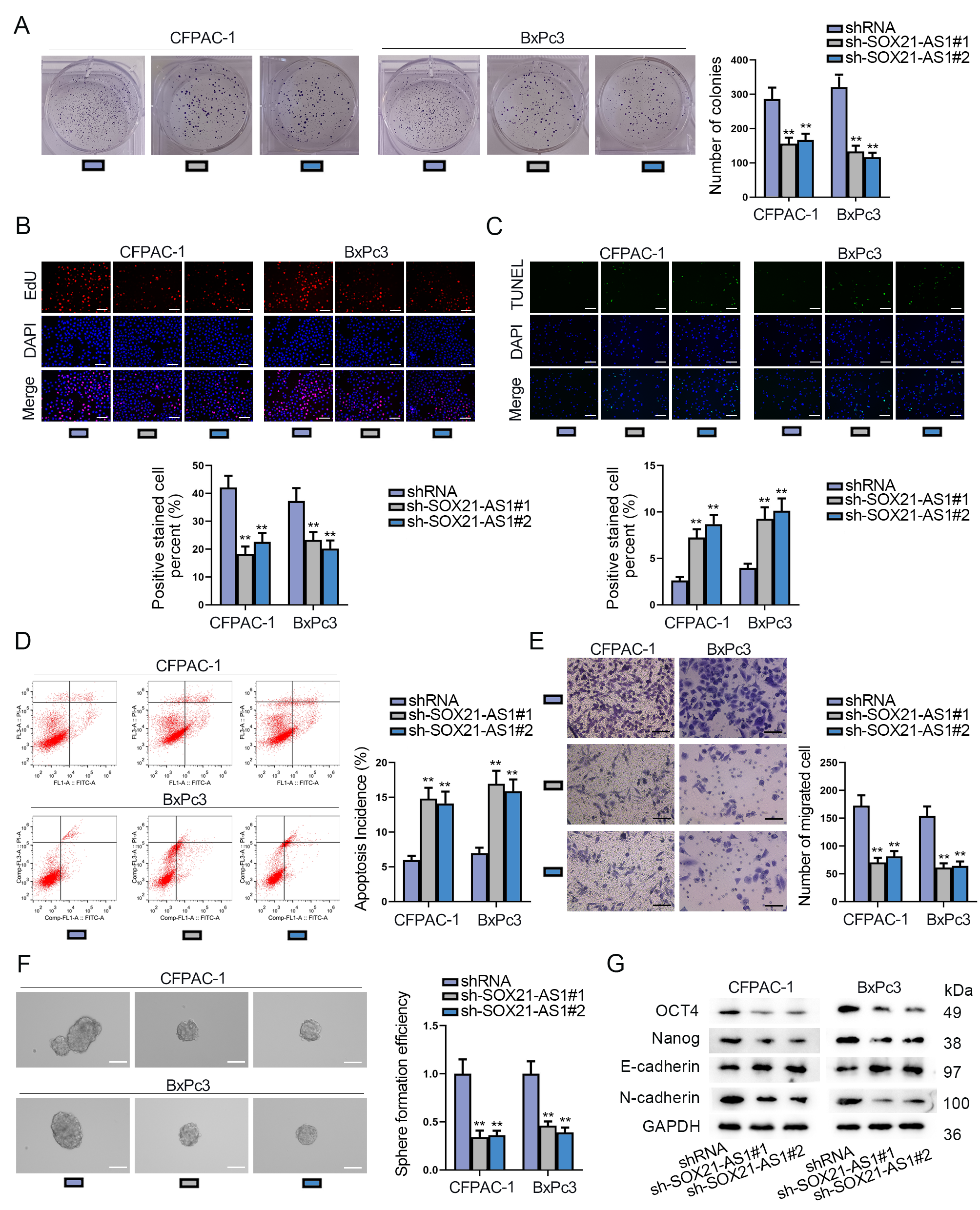

Supplement: Supplementary file 2 — Additional file 2: Figure S2. SOX21-AS1 silence suppressed the progression of PC. A-G Loss-of-function assays were performed in another two PC cell lines (CFPAC-1 and BxPc3) to further verify the malignant cell behaviors including proliferation, migration, EMT as well as apoptosis upon SOX21 silence treatment. **P < 0.01. [file 12967_2022_3521_MOESM2_ESM.tif]

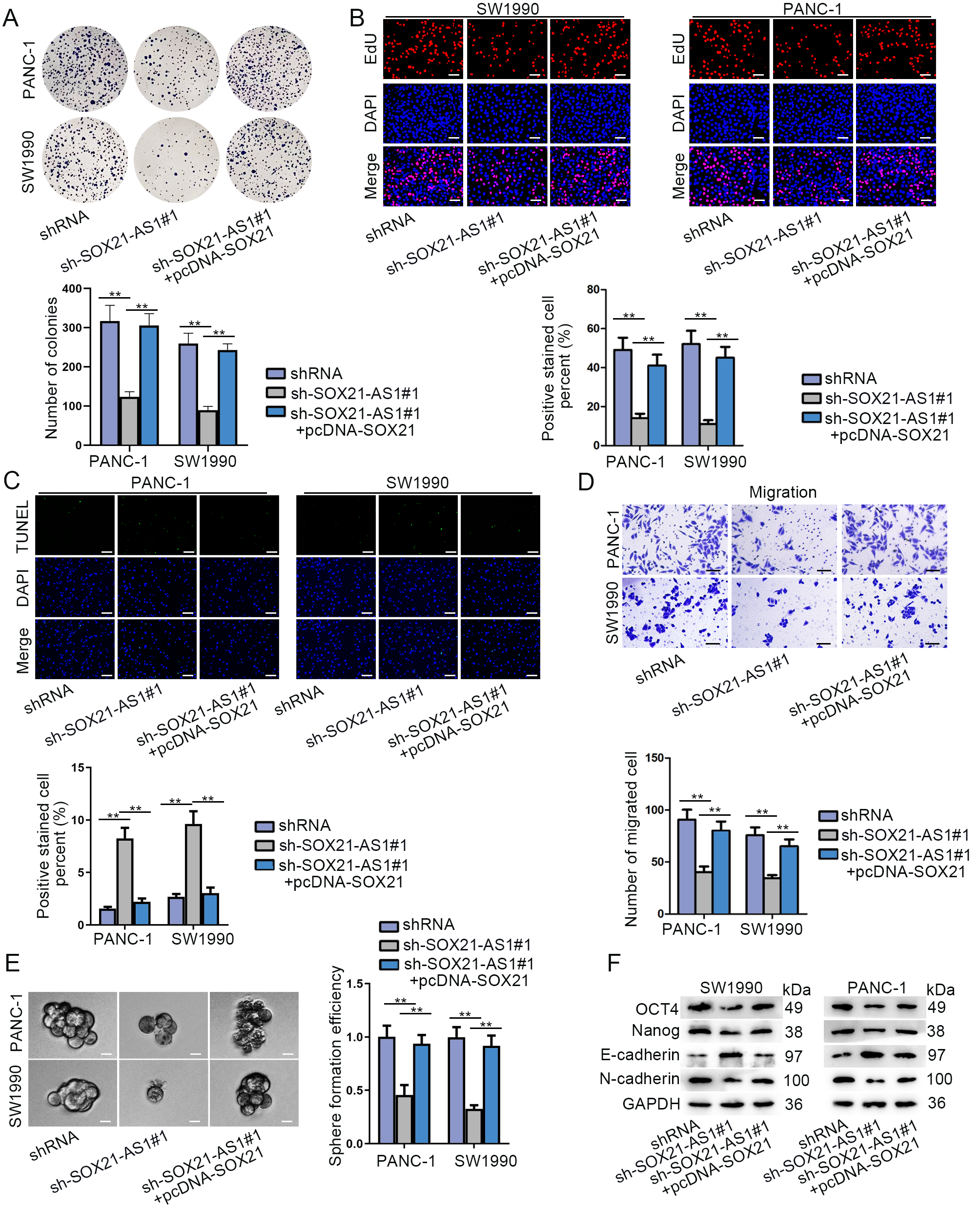

Supplement: Supplementary file 3 — Additional file 3: Figure S3. SOX21-AS1 affected PC cell proliferation, apoptosis, migration, stemness and EMT via modulating SOX21 expression. Rescue experiments in PC cells transfected with shRNA, sh-SOX21-AS1#1 and sh-SOX21-AS1#1 + pcDNA-SOX21, respectively. A-B Cell proliferation detection. C Cell apoptosis detection. D Transwell assays detected the migration ability. E Sphere formation assays detected the stemness. F Western blot analyzed the protein levels of EMT markers and transcription factors. **P < 0.01. [file 12967_2022_3521_MOESM3_ESM.tif]

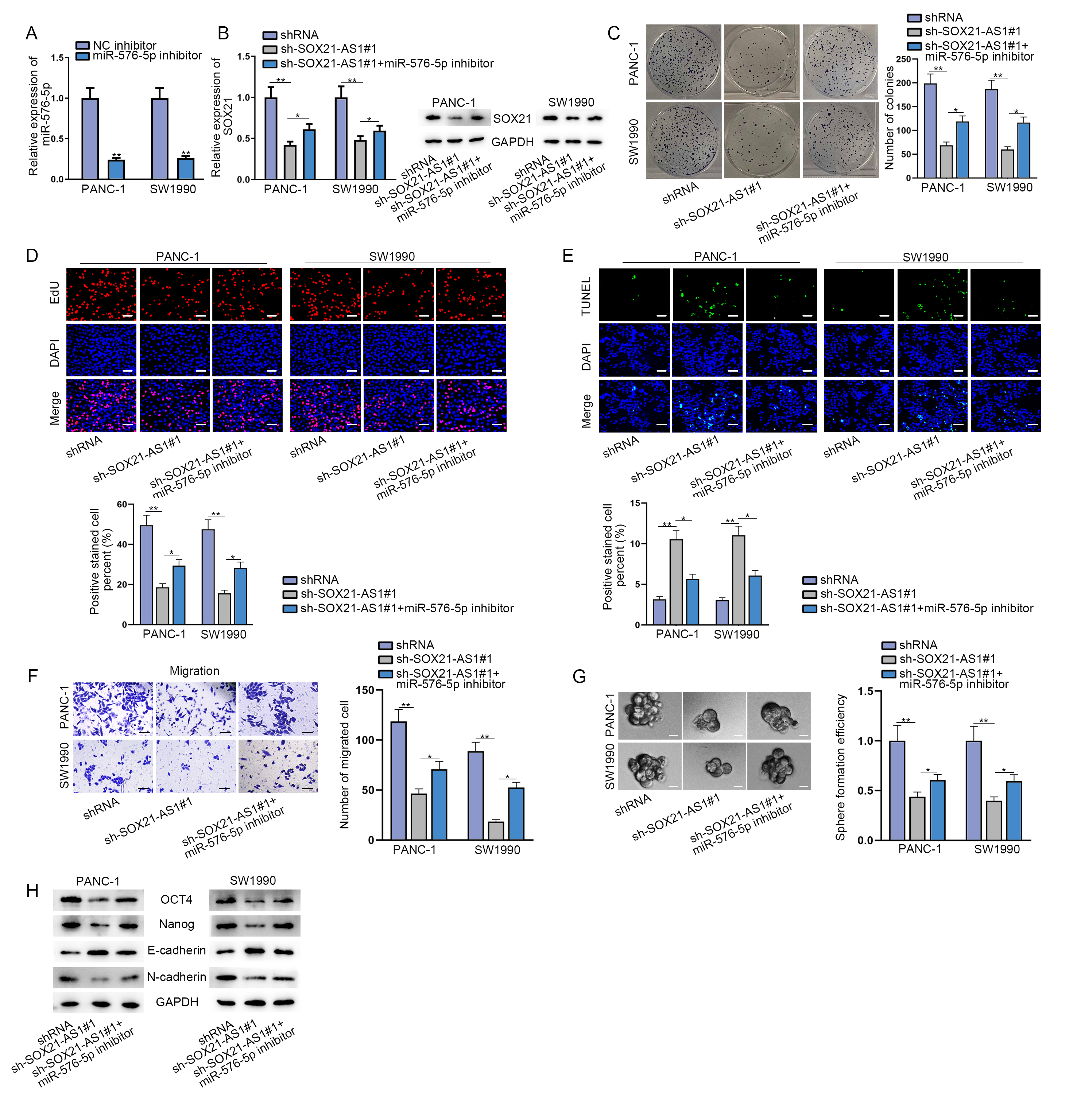

Supplement: Supplementary file 4 — Additional file 4: Figure S4. SOX21-AS1 affected PC cell proliferation, apoptosis, migration, stemness and EMT via interacting with miR-576-5p. A MiR-576-5p expression was decreased in PC cells. Rescue experiments were conducted in PC cells transfected with shRNA, sh-SOX21-AS1#1 and sh-SOX21-AS1#1 + miR-576-5p inhibitor, respectively. B SOX21 mRNA along with protein levels. C, D Cell proliferation detection. E TUNEL assays detected the cell apoptosis. F The migration of PC cells was testified through Transwell assays. G Sphere formation assays detected the stemness. H Western blot analyzed the protein levels of EMT markers and transcription factors. *P < 0.05, **P < 0.01 [file 12967_2022_3521_MOESM4_ESM.tif]
